# htSNPer1.0: software for haplotype block partition and htSNPs selection

**DOI:** 10.1186/1471-2105-6-38

**Published:** 2005-03-01

**Authors:** Keyue Ding, Jing Zhang, Kaixin Zhou, Yan Shen, Xuegong Zhang

**Affiliations:** 1 National Laboratory of Medical Molecular Biology; Institute of Basic Medical Sciences, Chinese Academy of Medical Sciences (CAMS) and Peking Union Medical College (PUMC), Beijing 100005, China; 2 MOE Key Laboratory of Bioinformatics/Department of Automation, Tsinghua University, Beijing 100084, China; 3 Chinese National Human Genome Center, Beijing 100176, China

## Abstract

**Background:**

There is recently great interest in haplotype block structure and haplotype tagging SNPs (htSNPs) in the human genome for its implication on htSNPs-based association mapping strategy for complex disease. Different definitions have been used to characterize the haplotype block structure in the human genome, and several different performance criteria and algorithms have been suggested on htSNPs selection.

**Results:**

A heuristic algorithm, generalized branch-and-bound algorithm, is applied to the searching of minimal set of haplotype tagging SNPs (htSNPs) according to different htSNPs performance criteria. We develop a software htSNPer1.0 to implement the algorithm, and integrate three htSNPs performance criteria and four haplotype block definitions for haplotype block partitioning. It is a software with powerful Graphical User Interface (GUI), which can be used to characterize the haplotype block structure and select htSNPs in the candidate gene or interested genomic regions. It can find the global optimization with only a fraction of the computing time consumed by exhaustive searching algorithm.

**Conclusion:**

htSNPer1.0 allows molecular geneticists to perform haplotype block analysis and htSNPs selection using different definitions and performance criteria. The software is a powerful tool for those focusing on association mapping based on strategy of haplotype block and htSNPs.

## Background

Several recent genome-wide and experimental studies suggested that the genome consists of chromosome regions of strong inter-marker linkage disequilibrium (LD) (i.e., haplotype blocks) and has discrete boundaries defined by recombination hotspots [1-4]. There are a few common haplotypes of limited haplotype diversity within each haplotype block, which can be characterized by only a small number of haplotype tagging SNPs (htSNPs). Haplotype blocks and htSNPs have great implication for association-based mapping of disease genes, by significantly reducing the genotyping effort with only a modest loss of power [5]. A new genomic map (i.e., haplotype map) for characterizing the haplotype structure in human genome is now underway to speed up the searching for genes involved in complex diseases.

A range of operational definitions has been used to identify haplotype block structures [1,2,6,7], which can be roughly cataloged into three groups [19]. First, there are methods based on diversity in the sequence, such as those of Patil et al. [1] and Zhang et al. [7], which define blocks with low sequence diversity by some diversity measure. The second group is based on LD methods, such as that of Gabriel et al. [2], which defines blocks with generally high pairwise LD within blocks and low pairwise LD between blocks. Finally, there are methods that look for direct evidence of recombination, such as that of [6], using the four-gamete test developed by Hudson and Kaplan [8] and defining blocks as apparently recombination-free regions.

There is still no consensus on the performance criteria of htSNPs selection. Broadly, these criteria are categorized into two groups. One comprises the diversity criteria which evaluate the information captured from the original haplotype diversity [1,9], such as the α-percent coverage, requiring that the total frequencies of all haplotypes completely distinguished by the htSNPs set should be no less than α. The other group consists of the association-based criteria, concerned most directly with the issue of prediction – the ability of the reduced set H to detect unknown SNPs in the set A of all SNPs within the genome region of interest [10].

To provide molecular geneticists more convenience in analyzing haplotype block structures and in selecting htSNPs, we develop a computational tool, htSNPer1.0, with a graphical user interface (GUI) implementing the above algorithms for block structure partition and htSNPs selection.

## Implementation

htSNPer1.0 is a computer program with a GUI for characterizing the haplotype block structure and selecting htSNPs. The core algorithm is implemented in C++ language, and the graphic interface is coded in Java. The software is platform-independent.

Here, we will be concerned with haplotype block partition and htSNPs selection of unphased autosomal SNPs genotype data. For the block definitions that can directly handle unphased genotype data such as Gabriel et al. [2] and those based on pairwise LD [11], the unphased data are first partitioned into blocks over which there is sufficient restriction of haplotype diversity. Then, haplotypes are estimated approximately within each block (by EM algorithm). Finally, based on these estimated haplotypes, htSNPs are selected according to certain htSNPs performance criterion [10]. For those block definitions that can only handle phased haplotype data [1,6], haplotypes are estimated first (by EM algorithm) from unphased genotype data. Then block partition and htSNPs selection are both based on these estimated haplotypes.

### Haplotype estimation – EM algorithm

We apply the EM algorithm used by SNPHAP to estimate haplotypes from genotype data [18]. When the data consist of a large number of SNPs, the number of possible haplotype instances may become extremely large. In order to avoid this problem, the program starts from the first two SNPs and extends the solution by sequentially adding the rest SNPs. As each new SNP is added, the number of possible haplotypes is expanded considering all possible larger haplotypes. After EM algorithm estimating the posterior probabilities, the program deletes genotype assignments with posterior probability lower than 0.001. Then the posterior probabilities of the rest genotype assignments are recomputed.

We use the EM algorithm in SNPHAP because it is simple and fast, and can be easily integrated in our C++ code. There are other algorithms like HAPLOTYPER [15], PHASE [16] and PLEM [17] that are better studied and more widely used. However, one distinctive feature of htSNPer1.0 is to estimate haplotypes within each haplotype block. Within the blocks there is very limited haplotype diversity, so in such cases the algorithm in SNPHAP performs reasonably well. If one likes to do the haplotype phasing before block partition, he/she can use HAPLOTYPER [15], PHASE [16] or PLEM [17] to get more accurate estimation, and then input the estimated haplotypes to htSNPer1.0 to do the block partition and htSNP selection.

### Definitions for haplotype blocks

htSNPer has integrated four haplotype block definitions: chromosome coverage [1], average pairwise LD |*D*'| [11], estimated pairwise LD confidence limits [2] with minor modifications by Wall and Prichard [14], and no historical recombination [6].

1. Chromosome coverage [1]. A block is defined as a region in which the sum frequencies of common haplotypes (whose frequency is over a threshold, e.g. 0.05) is no less than a threshold. For this definition of blocks we apply a dynamic programming for haplotype partitioning [7]. We define a Boolean function *block *(*i*, *j*) = 1 if the consecutive SNPs from *SNP*_*i *_to *SNP*_*j *_can be defined as a block according to the above definition, and *block *(*i*, *j*) = 0 otherwise. Let *f *(*i*, *j*) be the size of the minimal htSNP set found by GBB algorithm (see below) for α-percent coverage within the block from *SNP*_*i *_to *SNP*_*j*_. Given a block partition (1, *i*_1_), (*i*_1 _+ 1, *i*_2_),..., (*i*_*n*-1 _+ 1, *i*_*n*_), the total number of htSNPs for these *n *blocks is *f *(1, *i*_1_) + *f *(*i*_1 _+ 1, *i*_2_) +...+ *f *(*i*_*n*-1 _+ 1, *i*_*n*_). The optimal block partition is defined to be the one that minimizes the total number of htSNPs.

Denote *S*_*j *_to be the total number of htSNPs for the optimal block partition of the first *j *SNPs, and set *S*_0 _= 0. According to dynamic programming theory, we have . Through this recursion the dynamic programming partitions the haplotypes for the optimal block partition.

2. Average pairwise LD |*D*'| [11]. Within a block the average pairwise |*D*'| is no less than a threshold.

3. Estimated pairwise LD confidence limits [2] with minor modifications by Wall and Prichard [14]. For details see Additional file 1.

4. No historical recombination [6]. A block is defined as a region without any historical recombination, which is examined by Four Gamete Test.

The above definitions of 2, 3 and 4 do not guarantee a unique solution for partition. In htSNPer1.0, blocks are searched from the start of the input data and expanded as long as possible by sequentially adding the next SNPs.

### htSNPs selection criteria

htSNPer1.0 can find the minimal htSNP set of global optimum. Different definitions of optimum can be derived according to different htSNP performance criteria [10]. A generalized definition of "optimum" can be described as the minimal set of htSNPs that satisfies a given htSNP performance criterion. For example, weighted-average haplotype *r*^2 ^is regarded as one of the most informative association-based htSNP performance measure by Weale et al. [10], which is defined as following:

Weighted-average haplotype



where



and we denote  as the the frequency of haplotypes with allele 1 at SNP *i*,  as the frequency of haplotypes in the *g*th htSNP-defined group (haplotypes within each group are identical at htSNP loci), and  as the frequency of haplotypes both in the *g*th htSNP-defined group and with allele 1 at SNP *i*.

If the htSNP performance criterion is defined as the weighted-average haplotype *r*^2 ^of the selected set of htSNPs should be at least 90% of the maximum possible value (which is the weighted-average haplotype *r*^2 ^when all SNPs are selected as htSNPs), then the "optimum" according to this criterion can be described as the minimal set of htSNPs whose weighted-average haplotype *r*^2 ^is at least 90% of the maximum possible value.

We have integrated the three htSNP performance criteria into our htSNPer software: α-percent coverage [1], explained proportion of Clayton's haplotype diversity [9], and weighted-average haplotype *r*^2 ^[10].

α-percent coverage: the total frequencies of all haplotypes that are **not **completely distinguished by the htSNP set is less than 1 - α.

Explained proportion of Clayton's haplotype diversity: , where *f*_*i*_, *f*_*haplo *= *g *_and *f*_*i*,*g *_are defined in the same way as above.

Weighted-average haplotype *r*^2 ^: see above.

htSNPer1.0 takes advantages of a novel heuristic algorithm – Generalized Branch-and-Bound (GBB) algorithm, which is applicable for all kinds of htSNPs performance criteria, to search the minimal htSNPs set with both efficiency and global optimum, comparing to the exhaustive searching [7] which guarantees global optimum but runs very slowly, and to the greedy algorithm [1,13] which is faster but doesn't guarantee global optimum.

### The GBB algorithm

**Figure 1:**
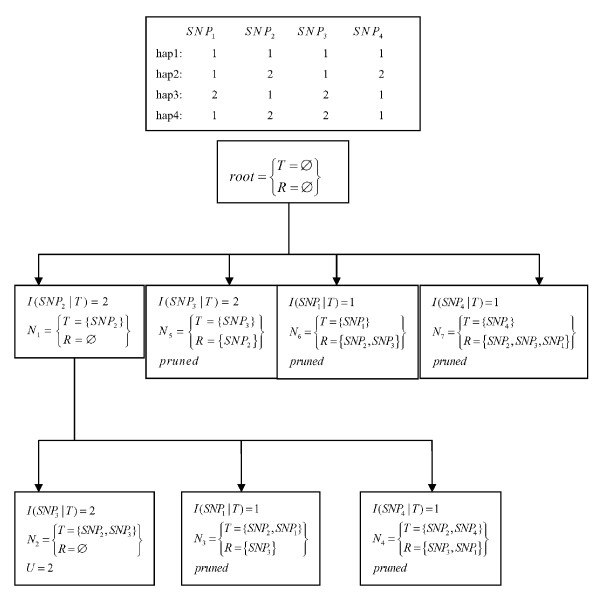
**An example for generialized branch-and-bounnd algorithm with 4 SNPs and 4 haplotypes**. The htSNPs performance criterion is to distinguish all the different haplotypes. The depth-first searching starts from *root*, exploring nodes in the order *N*_1_, *N*_2_,..., *N*_7_. *N*_2 _is the globally optimal solution. *N*_3_,..., *N*_7 _are all pruned from further consideration.

Consider a block B containing N haplotypes and each haplotype has M bi-allelic SNPs markers. Each SNP marker can divide N haplotypes into two groups: one consists of all the haplotypes with its major allele, and the other with its minor allele. GBB algorithm is based on the following branching rule and Generalized Prune-rule, using the depth-first searching strategy (Figure 1).

1) Each node {*T*, *R*} in the searching tree consists of two parts: the test-set *T *and the discard-set *R *where *T *is the set of SNPs that have been selected, and *R *is the set of SNPs that should not be selected for the future. If the set of all SNPs is denoted as *S*, then the set of SNPs that can be used at the node is *S*\(*T *∪ *R*). The search tree starts from the root node for which *T *= Φ and *R *= Φ.

2) A child node is generated by adding a SNP to *T *according to the branching rule. The node is pruned if it meets the Generalized Prune-rule.

### Importance calculation

Given a certain node {*T*, *R*}, SNPs in *T *divide all the haplotypes into *t *non-overlapping groups  called equivalence classes. Any haplotypes that belong to the same group are identical at all SNP sites in *T*. A biallelic SNP divides all the haplotypes into two groups: *G*_*major *_and *G*_*minor*_. To evaluate the competence of the SNP, the *importance *of a SNP is defined by



### Branching rule

Given a node {*T*, *R*}, sort the SNPs in *S*\(*T *∪ *R*) according to the importance calculation non-increasingly: *I*(*SNP*_1 _|*T*) ≥ *I*(*SNP*_2 _|*T*) ≥ … ≥ *I*(*SNP*_|*S*|-|*T*|-|*R*| _|*T*), create the children {*T *∪ *SNP*_1_, *R*}, {*T *∪ *SNP*_2_, *R *∪ *SNP*_1_}, {*T *∪ *SNP*_3_, *R *∪ *SNP*_1 _∪ *SNP*_2_}, ..., {*T *∪ *SNP*_|*S*|-|*T*|-|*R*|_, *SNP*_*h*_}, and explore the children in this order.

### Generalized prune-rule

Check whether the SNP subset *T *meets the htSNP performance criterion. If it does, prune the node when |*T*| ≥ *U*, or update *U *when |*T*| <*U *; otherwise, prune the node when |*T*| ≥ *U *or |*S*| - |*T*| - |*R*| < 1 where *U *is the size of the best solution found so far.

The Importance Calculation in Branching rule is originally devised for the α-percent coverage criterion [1,7]. But it is also applicable for other criteria, although it may not be the best one. Actually, one can devise specific Branching-rule and Prune-rule according to specific htSNPs selection criterion in the GBB framework to achieve super efficiency and global optimization. The GBB framework and algorithm we proposed are applicable to all htSNP criteria, and are at least more efficient than enumeration.

## Results and discussion

htSNPer1.0 takes advantages of a novel heuristic algorithm, Generalized Branch-and-Bound algorithm. It is applicable for all kinds of htSNPs performance criteria. The algorithm is of high computational efficiency and it can reach the global optimum.

**Figure 2:**
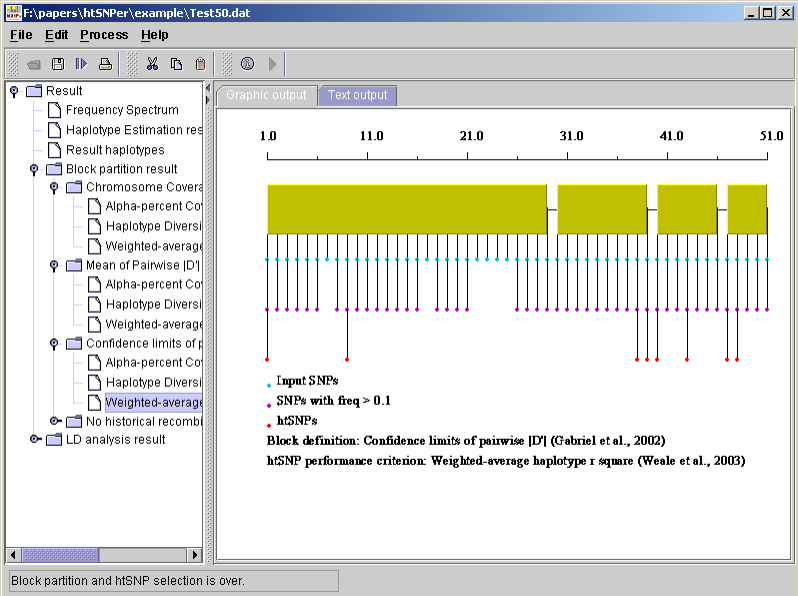
**Sample output from htSNPer1.0. **The first line showed the SNPs index, and could be replaced by the SNPs coordinate in its input. The brown color blocks represent the haplotype block structure in this region. Three classes of dots represent the input SNPs, the SNPs over a threshold (e.g., 0.10; defined in the optional dialogue), and the htSNPs, respectively.

htSNPer1.0 has integrated three htSNPs performance criteria and four haplotype block definitions. Besides genotype data, htSNPer1.0 can also handle haplotype data directly. It takes a simple flat-file as input. A dialogue box is used to set up parameters and for htSNPs selection algorithm (GBB algorithm and greedy algorithm). In the tabbed-output panel, htSNPer1.0 demonstrates the results both in the form of graphics and plain-texts. A graphical representation of haplotype block partition and htSNPs selection is provided in the graphic panel (Figure 2). In this example, there are 51 SNPs and 50 haplotypes in its input. The LD-based definition was used for haplotype partition, weighted-average haplotype *r*^2 ^for htSNPs performance criteria, and branch-and-bound algorithm in htSNPs selection. In the text-output panel, there is more information about the analysis and results, such as the methods/criterion used on haplotype block definition and htSNPs selection. Users can also select different haplotype definitions and htSNPs performance criteria to compare the results from the result tree in the left panel.

### Application example

#### Study sample

In order to compare the time used and the htSNPs numbers chosen with different softwares, we used the human chromosome 21 haplotype [1] as the test data. This dataset consists of 20 haplotype samples, and 24,047 common SNPs (minor allele frequency no less than 0.10). About 21% of the chromosome 21 data are missing data.

#### Results based on various haplotype block definitions and htSNP Selection Criteria

Results of the three different htSNPs performance criteria by htSNPer1.0:

Alpha-percent coverage: 3,953 blocks and 5,082 htSNPs.

Haplotype Diversity: 3,055 blocks and 4,619 htSNPs.

Weighted-average haplotype *r*^2 ^: with this criterion and GBB algorithm, htSNPer can not run on our computer because of the large amount of required memory. Using greedy algorithm instead of GBB (see Additional file 1), we get 3,098 blocks and 6,962 htSNPs.

Using the same data set, the same htSNPs performance criterion of α-percent coverage but the different block searching algorithm, Patil et al. [1] reported 4,135 blocks and 4,563 htSNPs. Zhang et al. [7] reported 2,515 blocks and 3,582 htSNPs. About 21% of the chromosome 21 data are missing data and different programs use different strategy to handle missing data. All these contribute to the differences between the results of the above programs.

We also ran the different programs to compare the computational efficiency. The algorithms used for comparison were the GBB algorithm in htSNPer1.0, the greedy algorithm by Zhang et al. [13] and the enumeration algorithm in Zhang et al. [7]. For comparison, we used the diversity-based haplotype block definition and α-percent coverage criterion in htSNPs selection (α = 0.8). Running on our computer with 2.4 GHz AMD Athlon processor (1GB memory), with the block definition of Patil et al. [1], α-percent coverage htSNPs performance criterion and a dynamic programming for haplotype partition, htSNPer1.0 requires 3 hours 23 minutes to identify 3,953 blocks and 5,082 htSNPs. For comparison, the greedy algorithm in Zhang et al. [13] requires 20.74 seconds, identifying 3,766 blocks and 8,733 htSNPs, and the enumeration method in Zhang et al. [7] was too slow to apply on our computer on this human chromosome 21 dataset. The difference in efficiency may be partially due to the different block partition searching strategies applied by these three programs. Zhang et al. [13] uses greedy algorithm for block partition, while htSNPer1.0 and Zhang et al. [7] use dynamic programming algorithm.

## Conclusion

In conclusion, htSNPer1.0 is a java-based program with Graphic User Interface. It allows molecular geneticists to perform haplotype block analysis and htSNPs selection using different definitions, different performance criteria, as well as different algorithms. The software is a powerful tool for those focusing on association mapping based on haplotype block and htSNPs strategy.

## Availability and requirements

htSNPer1.0 is a graphic user interface and a platform-independent software. The software is available at . The source code is available on request to the authors.

htSNPer1.0 takes a plain text file as input, either unphased autosomal SNPs genotype data or phased haplotype data. It requires the Java Running Environment (Jre1.4 or later version) to run the program properly. Detailed tutorials, htSNPer1.0 help system and examples are distributed within htSNPer1.0 software. Please inform the corresponding author if you are a non-academic user.

## Authors' contributions

K. Ding and J. Zhang provided software design, development and testing of the software, and J. Zhang wrote the source codes. K. Zhou participated in its design. Y. Shen and X. Zhang provided biological direction and validation of the tool. Y. Shen conceived of this project, and participated in its coordination. All authors have read and approved the final manuscript.

## Supplementary Material

Additional File 1 It provides more details of the methodology that are not covered in the main text. Click here for file
