# Supplementary material for: htSNPer1.0: software for haplotype block partition and htSNPs selection
Source: BMC Bioinformatics. 2005 Mar 1;6:38. doi: 10.1186/1471-2105-6-38 (PMC1274247; doi:10.1186/1471-2105-6-38)
Supplement: Additional File 1 — It provides more details of the methodology that are not covered in the main text. [file 1471-2105-6-38-S1.pdf]

Supplementary Material

of

htSNPer1.0: software for haplotype block partition and htSNPs selection

Keyue Ding, Jing Zhang, Kaixin Zhou, Yan Shen and Xuegong Zhang

Missing data

We apply the statistical strategy proposed by Anderson and Novembre (2003) for haplotype data. Suppose that the i th chromosome is missing data at the j th SNP. We fill in the missing data at $Y_{i,j}$ on the basis of a "window" of size w, as follows:

1. Identify all sets of w SNPs not missing in chromosome i such that each member of the set is separated from the j th SNP by no more than $w-1$ sites not missing in chromosome i. There will be, at most, $w+1$ such set (in cases in which j is near one of the ends of the string of SNPs in the data set there could be fewer than $w+1$). Denote the sites of the SNPs in such a set as J^+ (so there are $w+1$ or fewer J^+). These J^+ are "windows" of non-missing sites around j. Denote as K^1 the set of all chromosomes with allelic value 1, at site j, and for which all sites in J^+ have the same alleles that on chromosome i. Let K^2 be defined similarly for chromosomes with the allelic value 2 at j. For any chromosome k, let p_k denote the number of non-missing sites it has in J^+, and define P^1 as $\sum_{k \in K^1} p_k$ and P^2 as $\sum_{k \in K^2} p_k$.
2. For each of the $w+1$ (or fewer) sets J^+, calculate the pair $Q_1 = P^1/(P^1+P^2)$ and $Q_2 = P^2/(P^1+P^2)$, and denote by (Q_1^*, Q_2^*) the pair of those quantities that maximizes $|Q_1 - Q_2|$ over the different sets J^+.
3. Fill in the hole $Y_{i,j}$ with a 1 for probability Q_1^* and 2 for probability Q_2^*.

The above process scans all the "windows" of size $w+1$ (or fewer) for one with the most strong evidence to assign the missing data with 1 or 2. However, this method is recently proposed and fairly simple which needs further improving.

We also code the ***EM*** algorithm to handle the missing data by estimating the possible haplotypes frequencies and picking the ones with the largest posterior probabilities. Users can choose either one of these two methods.

Details about Haplotype block definition of Estimated pairwise LD confidence limits

Estimated pairwise LD confidence limits (Gabriel et al., 2002) with minor modifications by Wall and Pritchard (2003). Assuming that true allele frequencies equal the sample allele frequencies, for each pair of sites, confidence limits are determined by calculating the likelihood of the observed data as a function of $|D'|$: $l(|D'|) = \Pr(data \| D'|)$. The upper bound $C_U = \min_{l(|D'|)\geq 0.95}(|D'|)$, the lower bound $C_L = \max_{l(|D'|)\leq 0.05}(|D'|)$. Each pair of SNPs is classified into one of three categories: "strong LD" if $C_L \geq 0.7$ and $C_U \geq 0.98$; "historical evidence of recombination" if $C_U < 0.9$; "other" else. A region is defined as a block if it meets both the following: (1) the endpoint SNPs are in "strong LD" and (2) the number of pairs in "strong LD" is at least 19 times the number in "historical evidence of recombination". SNPs are not permitted to be members of more than one block. For diploid genotype data, $l(|D'|) = \Pr(data \| D'|)$ is calculated as following (Hudson, 2001).

$$\Pr(n_d) = \frac{n!}{\prod_{i=1}^{9} n_i!}(p_{11}^2)^{n_1}(2p_{11}p_{12})^{n_2}(p_{12}^2)^{n_3}(2p_{11}p_{21})^{n_4}$$
$$\times(2p_{11}p_{22}+2p_{12}p_{21})^{n_5}(2p_{12}p_{22})^{n_6}(p_{21}^2)^{n_7}(2p_{21}p_{22})^{n_8}(p_{22}^2)^{n_9}$$

Where $n_d = (n_1, n_2, ..., n_9)$ according to ***Table 1*** with $n = \sum_{i=1}^{9} n_i$, and p_{ij}, $i = 1,2; j = 1,2$, is

the frequency of the haplotype with A_i and B_j at two loci., which can be counted given $|D'| = 0.01\times k$, $p_{i\cdot}$, $p_{\cdot i}$, and $D' = \begin{cases} \dfrac{p_{11} - p_{1\cdot}p_{\cdot 1}}{\min(p_{1\cdot}p_{\cdot 2}, p_{2\cdot}p_{\cdot 1})} & p_{11} - p_{1\cdot}p_{\cdot 1} > 0 \\ \dfrac{p_{11} - p_{1\cdot}p_{\cdot 1}}{\min(p_{1\cdot}p_{\cdot 1}, p_{2\cdot}p_{\cdot 2})} & p_{11} - p_{1\cdot}p_{\cdot 1} < 0 \end{cases}$ as following:

$p_{11} = 0.01k \times \min(p_{1\cdot}p_{\cdot 2}, p_{2\cdot}p_{\cdot 1}) + p_{1\cdot}p_{\cdot 1}$ or

$p_{11} = -0.01k \times \min(p_{1\cdot}p_{\cdot 1}, p_{2\cdot}p_{\cdot 2}) + p_{1\cdot}p_{\cdot 1}$ and $p_{12} = p_{1\cdot} - p_{11}$, $p_{21} = p_{\cdot 1} - p_{11}$,

$p_{22} = 1 - p_{1\cdot} - p_{\cdot 1} + p_{11}$. So $\Pr(n_d \,\|\, D'| = 0.01k)$ can be counted for $k = 0,1,...100$.

Table 1: Notation for numbers of observed two-locus diploid genotypes

	B_1/B_1	B_1/B_2	B_2/B_2
A_1/A_1	n_1	n_2	n_3
A_1/A_2	n_4	n_5	n_6
A_2/A_2	n_7	n_8	n_9

For haplotype data, we use the following equation to calculate the likelihood of the observed data,

$\Pr(n_h) = \dfrac{n!}{\prod_{\substack{i=1,2\\ j=1,2}} n_{ij}} \prod_{\substack{i=1,2\\ j=1,2}} p_{ij}^{n_{ij}}$ where $n_h = (n_{11}, n_{12}, n_{21}, n_{22})$ with $n = \sum_{\substack{i=1,2\\ j=1,2}} n_{ij}$. Other calculations are the same with genotype data. (***Handbook of Statistical Genetics***, Balding et al., 2001).

Pairwise |D'| pattern

htSNPer also provides LD pattern of pairwise |D'| since it is directly related with block definitions of 2, 3 and 4. For genotype data, we apply EM algorithm to estimate the four haplotype frequencies of pairwise loci (Hudson, 2001).

BB-based greedy algorithm

htSNPer also provides a BB-based greedy algorithm which is based on the GBB algorithm. The only difference is that in branching step greedy algorithm only explores the child with $\{T \cup SNP_1, R\}$, which adds the most important SNP to T, and discards all the other children nodes.

This greedy algorithm doesn't guarantee to find the minimal set of htSNPs, but much faster than GBB algorithm.

REFERENCES

Hudson, R.R. (2001) Linkage disequilibrium and recombination. In Balding, D.J., Bishop, M. and Cannings, C. (eds), *Handbook of Statistical Genetics*. Wiley, New York, pp. 309-324.

Anderson, E.C., and Novembre, J. (2003) Finding Haplotype Block Boundaries by Using the Minimum-Description-Length Principle. *Am. J. Hum. Genet.*, **73**, 336-354.

De Bontridder, K.M.J., Lageweg, B.J., Lenstra, J.K., Orlin, J.B., Stougie, L. (2002) Branch-and-bound algorithms for the test cover problem. Algorithms—ESA 2002, LNCS, Springer, Berlin, 223-233.

Gabriel, S.B., Schaffner, S.F., Nguyen, H, Moore, J.M., Roy, J., Blumenstiel, B., Higgins, J., DeFelice, M., Lochner, A., Faggart, M., et al. (2002) The structure of haplotype blocks in the human genome. *Science*, **296**, 229-232.

Garey, M.R. and Johnson D.S. (1979) Computers and Intractability (Feeman, New York), p. 222

Jeffreys, A.J., Kauppi, L., and Neumann, R. (2001) Intensely punctuate meiotic recombination in the class II region of the major histocompatibility complex. *Nat. Genet.*, **29**, 217-222.

Johnson, G.C.L., Esposito, L., Barratt, B.J., Smith, A.N., Heward, J., Di Genova, G., Ueda, H., Cordell, H.J., Eaves, I.A., Dudbridge, F., et al. (2001) Haplotype tagging for the identification of common disease genes. *Nat Genet.,* **29**, 233-237.

May, C.A., Shone, A.C., Kalaydjieva, L., Sajantila, A., and Jeffreys, A.J. (2002) Crossover clustering and rapid decay of linkage disequilibrium in the Xp/Yp pseudoautosomal gene SHOX. *Nat. Genet.*, **31**, 272-275.

Patil,N., Berno,A.J., Hinds,D.A., Barrett,W.A., Doshi,J.M., Hacker,C.R., Kautzer,C.R., Lee,D.H., Marjoribanks,C., McDonough,D.P. et al., (2001) Blocks of limited haplotype diversity revealed by high-resolution scanning of human chromosome 21. *Science,* **294**, 1719-1723.

Reich DE, Cargill M, Bolk S, Ireland J, Sabeti PC, Richter DJ, Lavery T, Kouyoumjian R, Farhadian SF, Ward R, Lander ES (2001) Linkage disequilibrium in the human genome. Nature 411:199–204

Wang, N., Akey, J.M., Zhang, K., Chakraborty, R., and Jin, L. (2002) Distribution of recombination crossovers and the origin of haplotype blocks: the interplay of population history, recombination, and mutation. *Am. J. Hum. Genet.,* **71**, 1227-1234.

Weale, M.E., Depondt, C., Macdonald, S. J., Smith, A., Lai, P.S., Shorvon, S.D., Wood, N.W., and Goldstein, D.B. (2003) Selection and Evaluation of Tagging SNPs in the Neuronal-Sodium-Channel Gene SCN1A: Imploications for Linkage-Disequilibrium Gene Mapping. *Am. J. Hum. Genet.*, **73**, 551-565.

Zhang, K., Deng, M., Chen, T., Waterman,M.S. and Sun,F. (2002) A dynamic programming algorithm for haplotype block partition. *Proc. Natl. Acad. Sci. USA*, **99** 7335-7339.

http://www-gene.cimr.cam.ac.uk/clayton
